# Circumstances of provoked recurrent venous thromboembolism: the Austrian study on recurrent venous thromboembolism

**DOI:** 10.1007/s11239-019-01965-z

**Published:** 2019-10-17

**Authors:** Hannah C. Puhr, Lisbeth Eischer, Hana Šinkovec, Ludwig Traby, Paul A. Kyrle, Sabine Eichinger

**Affiliations:** 1 Department of Medicine I, Medical University of Vienna, Waehringer Guertel 18-20, 1090 Vienna, Austria; 2 Center for Medical Statistics, Informatics and Intelligent Systems, Medical University of Vienna, Vienna, Austria; 3 Karl Landsteiner Institute of Clinical Thrombosis Research, Vienna, Austria

**Keywords:** Embolism, Recurrence, Risk factors, Thromboembolism, Thrombosis

## Abstract

Patients with unprovoked deep-vein thrombosis (DVT) of the leg or pulmonary embolism (PE) have a high recurrence risk. How often these recurrences are provoked by a temporary risk condition is unknown. In a cohort of patients with unprovoked venous thromboembolism (VTE), we evaluated the clinical circumstances of recurrence. We studied patients with DVT of the leg and/or PE. End point was recurrence of objectively verified symptomatic VTE. Provoked recurrence was defined according to guidance criteria. 1188 patients were followed for a median of 8.9 years after withdrawal of oral anticoagulants. 312 patients had recurrent VTE, which was provoked in 42 (13%). Recurrence was related to a major risk factor in 19, to a minor risk factor in 22, and to a persistent risk factor in one patient(s). 14 recurrences occurred after major surgery and 5 during hospitalization. Ten recurrences occurred after minor surgery, eight after trauma and three during female hormone intake. Four recurrences occurred during heparin prophylaxis. The incidence of provoked VTE recurrence appears to be low. VTE can recur when prevention is stopped or even during thromboprophylaxis. Surgery and trauma are frequent risk factors.

## Highlights

- Patients with unprovoked venous thromboembolism (VTE) have a high recurrence risk.
- 42 of 1188 patients with a first unprovoked VTE had a provoked recurrence.
- Surgery and trauma were the most prevalent risk factors.
- Recurrent VTE was mostly diagnosed when thromboprophylaxis is no longer sustained.

## Introduction

Venous thromboembolism (VTE), which encompasses deep-vein thrombosis (DVT) and pulmonary embolism (PE), is a frequent and potentially fatal disease. In a large proportion of patients, VTE occurs as the consequence of temporary risk conditions, with surgery, hospitalization, trauma or immobilization as the most important ones [1]. In a study from Norway, for instance, a first VTE was related to such an acquired risk factor in approximately 50% of patients [2].

VTE is a preventable disease as pharmacologic thromboprophylaxis is highly effective. A history of VTE is regarded as a major risk factor for VTE recurrence, particularly when a patient is exposed to a temporary risk condition. Under these circumstances, guidelines recommend standard pharmacological thromboprophylaxis for surgical as well as for non-surgical patients [3, 4]. Whether these measures are equally effective in patients with a previous VTE compared with patients with no history of VTE is unknown.

In the Austrian Study of Recurrent Venous Thromboembolism (AUREC), we followed a large cohort of patients with a first unprovoked DVT of the leg and/or PE over many years [5]. We found that this patient population has a recurrence risk of almost 50% after 20 years [6]. In this analysis we set out to evaluate the clinical circumstances under which these recurrences occurred, i.e. we were interested in the proportion of patients who had a recurrent VTE triggered by a temporary risk factor (“provoked”) compared to those with unprovoked VTE, and in the determinants of such provoked VTE recurrences.

## Materials and methods

### Patients and study design

AUREC is an ongoing prospective cohort study involving four thrombosis centers in Vienna. Details of the study design were published recently [5]. Between July 1992 and September 2008, patients older than 18 years of age who had been treated with oral anticoagulants for at least 3 months after a first unprovoked DVT of the leg or PE were enrolled after providing written informed consent.

Patients with an index DVT of the leg or PE secondary to surgery, trauma, immobilization, pregnancy or cancer, patient requiring indefinite anticoagulation for other reasons than VTE and patients with major thrombophilia were excluded. Major thrombophilia was defined as deficiency of antithrombin, protein C, or protein S; presence of the lupus anticoagulant; homozygosity for the factor V Leiden mutation or the factor II G20210A mutation, double heterozygosity for the two mutations, or active cancer. The day of discontinuation of oral anticoagulants was defined as the day of study entry. Patients were provided with detailed written information on the symptoms of VTE and were instructed to report to one of the thrombosis centers in case of symptoms. All women were strongly discouraged from using contraceptive pills or hormone-replacement therapy. All patients were instructed to inform their treating physicians on their history of VTE in order to receive standard thromboprophylaxis in case of surgical or non-surgical thrombotic risk conditions. Patients were observed at three-month intervals for the first year and every 6 months thereafter.

### Diagnosis of venous thromboembolism

The diagnosis of DVT was established by color-coded duplex sonography or venography. The diagnosis of PE was made by computed tomography angiography or by ventilation–perfusion lung scanning. Patients with both DVT and PE were categorized as having PE.

### Outcome measures

The endpoint of the study was recurrence of symptomatic VTE confirmed by color-coded duplex sonography or venography, computed tomography angiography or ventilation–perfusion lung scanning. DVT was considered to have recurred if the patient had: a thrombus in the leg not affected by the previous thromboembolic event; a thrombus in another deep vein in the same leg as the previous event; or a thrombus in the same venous system as the previous event with proximal extension of the thrombus (if the upper limit of the original thrombus had been visible) or with a constant filling defect surrounded by contrast medium (if the original thrombus had not been visible). Recurrent PE was defined as a central filling defect of one or more pulmonary arteries with or without a thin rim of contrast material surrounding the filling defect on computed tomography or was diagnosed according to the criteria of the Prospective Investigation of Pulmonary Embolism Diagnosis in the case of ventilation-perfusion lung scanning [7].

Provoked recurrence was defined according to International Society of Thrombosis and Haemostasis guidance criteria as follows [8]. “Major”: VTE within 3 months after a surgery with general anesthesia for greater than 30 min, hospitalization (only ‘bathroom privileges’) for at least 3 days with an acute illness; “Minor”: VTE within 2 months after surgery with general anesthesia for less than 30 min., admission to hospital for less than 3 days with an acute illness, estrogen therapy, or leg injury associated with reduced mobility for at least 3 days.

In case of provoked recurrent VTE the circumstances of the event (triggering factor, treatment, type and duration of thromboprophylaxis, duration of hospitalization) were obtained by face-to-face patient interview, telephone conversations, and standardized questionnaire and/or were retrieved from medical records.

### Statistical analysis

Continuous variables were described by median and interquartile range (IQR) and categorical variables by absolute frequency and percentage. Median and quartiles of the full study cohort’s follow-up distribution were estimated by the reverse Kaplan–Meier method. Kaplan–Meier method was used to estimate the cumulative probability of recurrent VTE. Patients were censored at the time of withdrawal, if they left the study, or at the time of their last visit, if they were lost to follow-up. Patients that died or re-initiated antithrombotic treatment were also considered as censored. The characteristics of patients with provoked and those with unprovoked VTE were described by means of descriptive statistics. No statistical tests were used to compare both groups as adjusting for an event that might have triggered a recurrence would be necessary, an information that was only available for those patients that indeed experienced a recurrence during the follow-up.

## Results

### Study population

The characteristics of the 1188 study patients with a first unprovoked VTE are depicted in Table 1. The median age was 48.6 years and 46% were male. They had been treated with oral anticoagulants for a median of 6.5 (IQR 6.1–7.7) months and were prospectively followed in median for 8.9 years (IQR 3.3–12.8).

**Table 1:** Baseline characteristics of 1188 patients with first unprovoked VTE and of 312 patients with recurrent VTE according to presence or absence of a provoking factor; values are absolute frequencies (percentage) and median (interquartile range), respectively

Characteristics	unprovoked first VTE (n = 1188)	provoked recurrence (n = 42)	unprovoked recurrence (n = 270)
Sex—m/f, n (%)	550 (46)/638 (54)	22 (52)/20 (48)	195 (72)/75 (28)
Age (years)	48.6 (37.2–60.7)	51.2 (42.2–57.9)	52.1 (42.1–61.5)
Location of initial VTE, n (%)			
Distal DVT	237 (20)	5 (12)	35 (13)
Proximal DVT	463 (39)	15 (36)	122 (45)
PE	488 (41)	22 (52)	113 (42)
Duration of anticoagulation (months)	6.5 (6.1–7.7)	6.7 (6.1–8.7)	6.6 (6.1–7.9)
Observation time (months)	107 (39.8–153.2)^a^	43.1 (13.6–72.5)	39.7 (12.4–82.3)
Body mass index (kg/m^2^)	26.4 (23.5–29.7)	28.7 (25.4–31.4)	27.4 (24.8–30.1)
Factor V Leiden, n (%)	299 (25)	7 (17)	83 (31)
Factor II G20210A, n (%)	61 (5)	21 (8)	2 (5)

^a^Estimated by the reverse Kaplan–Meier method

548 patients were censored during follow-up for the following reasons: 253 (21%) were started on long-term antithrombotic treatment for reasons other than VTE [aspirin in 194 (16%) patients, oral anticoagulants in 59 (5%) patients]; 72 (6%) became pregnant and received thromboprophylaxis with a low-molecular-weight heparin; 31 (3%) died for reasons other than recurrent PE; 51 (4%) patients received a diagnosis of cancer. 48 (4%) patients withdrew their consent. 93 (8%) patients were lost-to-follow-up.

### Recurrent venous thromboembolism

A total of 312 (26%) of 1188 patients had recurrent VTE. The probability of recurrence after 1 year was 6.7% (95%CI 5.2–8.1%), after 5 years 19.8% (95%CI 17.3–22.3%) and after 10 years 30.9% (95%CI 27.6–34%). The recurrence was provoked in 42 (13%) patients and unprovoked in 270 (87%) patients. 5 (0.4%) patients had fatal PE; all of those were unprovoked. The characteristics at study entry of patients with provoked and unprovoked recurrence are depicted in Table 1. The proportion of men and women was similar among patients with provoked recurrence whereas men had more frequently an unprovoked recurrence. The frequency of factor V Leiden was lower in patients with provoked recurrence (17% vs. 31%). The characteristics of patients with provoked recurrence were similar to those with unprovoked recurrence regarding age, location of initial VTE, duration of anticoagulation, observation time, body mass index, or presence of factor II G20210A.

### Circumstances of provoked VTE recurrence

Of 42 provoked recurrences, 22 (52%) were a DVT of the leg [distal DVT in 10 (24%) and proximal DVT in 12 (29%) patients], 19 (45%) patients had an isolated PE and 1 (2%) patient had a DVT in a subclavian vein. The site of recurrent VTE was similar in patients with provoked and unprovoked recurrence: of the 270 patients with unprovoked recurrence, 49 (18%) patients had distal DVT, 96 (36%) patients had proximal DVT, 121 (45%) patients had an isolated PE and 4 (2%) patients had DVT at another site.

As regards patients with provoked recurrence, the site of initial and recurrent VTE was the same in 24 (57%) patients (16 patients had PE and 8 patients had DVT at presentation as well as at recurrence) and was different in 18 patients (43%). Recurrent VTE was related to a major risk factor in 19 (45%) patients, to a minor risk factor in 22 (52%) patients, and to a persistent risk factor (cancer) in one patient (Table 2).

**Table 2:** Determinants of provoked recurrence

Provoking factor	n	Sex (m/f)	Age (years), median (IQR)	Interval between provoking factor and diagnosis of VTE (days), median (IQR)
Major				
Surger, > 30 min anesthesia^a^	14	4/10	52.7 (39.8–56.0)	30.5 (8.3–47.8)
Hospitalization, confined to bed > 3 days^b^	5	4/1	52.9 (50.9–53.4)	during hospital stay
Minor				
Surgery, < 30 min anesthesia^c^	10	5/5	54.9 (43.4–57.9)	12.0 (7.3–17.8)
Estrogen therapy	3	0/3	35.5 (28.6–40.7)	unknown
Acute illness^d^	1	1/0	42.4	during hospital stay
Trauma^e^	8	7/1	56.7 (43.4–66.7)	7.0 (7.0–18.0)^f^
Persistent				
Cancer	1	1/0	49.1	unknown

^a^Total hip replacement [n = 1], total knee replacement [n = 1], foot surgery [n = 2], salivary gland excision [n = 1], laparoscopic cholecystectomy [n = 1], total colectomy [n = 1], oncocytoma excision [n = 1], abdominoplasty [n = 1], oral surgery [n = 1], vein surgery [n = 1], knee surgery [n = 1], hysterectomy [n = 2]

^b^Pneumonia [n = 1], sepsis [n = 1], diarrhea [n = 1], vestibularis neuritis [n = 1], brain injury [n = 1]

^c^Skin excision [n = 1], curettage [n = 2], arthroscopy [n = 4], cystoscopy [n = 1], lithotripsy [n = 1], prostate biopsy [n = 1]

^d^Myocardial infarction [n = 1]

^e^Rib fractures [n = 2], injuries of the lower leg [n = 6]

^f^Data missing for 1 patient

Of the 19 recurrences related to a major risk factor, 14 occurred after major surgery and 5 during hospitalization for a period longer than 3 days. In the 14 patients with a VTE recurrence after major surgery, the median time interval between surgery and diagnosis of recurrence was in median 30.5 days with a maximum of 61 days. VTE after major surgery was more often seen in women than in men (10 vs. 4). Of the 22 recurrences associated with a minor risk factor, 10 occurred after minor surgery, 8 after trauma, 3 during female hormone intake, and 1 was related to an acute illness. In the 10 patients with a VTE recurrence after minor surgery, the time interval between surgery and diagnosis of recurrence was in median 12 days with a maximum of 61 days. The median time interval between trauma and diagnosis of recurrence was 7 days with a maximum of 42 days. VTE after trauma was more frequent among men than women (7 vs. 1).

In our study, patients received postoperative pharmacological thromboprophylaxis according to national or international guidelines. Nevertheless, 4 patients had recurrence despite thromboprophylaxis with a low-molecular-weight heparin: one patient 2 days after brain injury, and three patients after surgery (2 days after abdominoplasty, 1 week after arthroscopy and 1 week after hysterectomy, respectively).

## Discussion

The most important finding of this analysis is that the vast majority of patients who experienced recurrence had an unprovoked VTE recurrence, whereas recurrence related to a temporary risk condition was infrequent and occurred in only 42 of the 1188 study patients.

VTE is a preventable disease as both primary and secondary thromboprophylaxis are very effective [9]. Patients with a history of VTE are regarded at high risk of recurrence when exposed to a temporary risk factor and guidelines recommend pharmacologic thromboprophylaxis under such circumstances [3, 4]. At entry into our study, patients were informed about the increased thrombosis risk during circumstantial risk conditions and the need for thromboprophylaxis. They were also instructed to inform their treating physicians on their VTE history. Female patients were strongly discouraged from using contraceptive pills or hormone-replacement therapy. Our observation of a low number of provoked VTE recurrences supports our strategy of guideline consideration and intensive patient counseling.

Our findings also show that VTE recurrence cannot completely be prevented when these high-risk patients are exposed to a temporary risk factor. Not unexpected, surgery and trauma were the two most frequent risk factors for recurrent VTE accounting for almost two-thirds of recurrences. These findings corroborate those of the Dutch MEGA follow-up study, in which patients with a history of DVT, who required lower-leg cast, were found to have higher risk of recurrence compared with patients without the need for a cast [10].

Some interesting lessons can be learned from our observations. Recurrent VTE was diagnosed in median 31 days after major surgery, 12 days after minor surgery, and 7 days after trauma, i.e. at the time when thromboprophylaxis is no longer sustained in many of these patients. Most probably, these late recurrences may have been prevented by prolonged thromboprophylaxis.

Four patients had a recurrent VTE event despite ongoing thromboprophylaxis with low-molecular-weight heparin supporting the notion that primary thromboprophylaxis is not 100% effective. Whether these events could have been prevented by a higher heparin dose, and if so, at what risk of bleeding, is unclear. How patients who might benefit from such a strategy can be identified, also requires more research.

At study entry, women were strongly discouraged from using female hormones and we trust that almost all of them followed this advice. Nevertheless, we recorded a VTE recurrence in 3 women who restarted female hormones. This observation once again underlines the importance of intensive counseling on the risk of VTE recurrence.

In contrast to the patient population with unprovoked recurrence in whom the likelihood of recurrence is much higher among males than females [11], this was not the case in patients with provoked recurrence. One explanation could be that the strength of the triggering circumstantial risk factor outweighed the risk conferred by male gender thereby masking the gender effect. In a sub-analysis, we found a preponderance of females among patients with VTE after major surgery and more males among patients with VTE after trauma. However, this could very well be a chance finding, as patient numbers were small. Our data do not justify a distinction between men and women regarding dose and duration of thromboprophylaxis.

In the majority of patients, the site of the initial and of the recurrent VTE is the same [12]. Interestingly, in the subpopulation of patients with recurrent provoked VTE, we observed the same phenomenon: 16 patients had isolated PE and eight patients had DVT both at presentation as well as at recurrence. Conversely, the site of VTE at study entry and at recurrence was different in only less than half of patients. We believe that this observation may have some clinical relevance as mortality and morbidity of PE greatly exceeds that of DVT. [13]

Neither the factor V Leiden mutation nor the G20210A mutation in prothrombin gene confers a clinically relevant increase in the risk of recurrent VTE [12, 14–16]. In patients with provoked recurrence the proportion of carriers of the factor V Leiden mutation and of the prothrombin mutation was similar or even lower compared to that found in cohorts with unprovoked (recurrent) VTE [17]. This supports the concept that knowledge about the presence or absence of laboratory thrombophilia is not helpful when assessing the risk of recurrent VTE during a temporary risk condition, in particular before surgery or after trauma.

Strengths and limitations need to be considered. We followed a large cohort of patients, in total almost 1.200, over a very long period of time, in median 8.9 years. The patient population was homogenous: all patients had unprovoked VTE at presentation and were therefore at high risk of recurrence. Both the initial event as well as recurrence was objectively diagnosed in all patients. Categorization of patients with provoked VTE recurrence as having VTE triggered by a major, a minor, or a persistent risk factor was performed according to a recent guidance document of the Scientific and Standardizing Committee of the International Society on Thrombosis and Haemostasis [8]. The circumstances of the event such as triggering factor, treatment, type and duration of thromboprophylaxis, and the duration of hospitalization were reliably documented in the majority of patients. The most important limitation is that we are not aware of type and circumstances of the many temporary risk situations our patients were exposed to and hence those patients who did not experience provoked recurrent VTE. We can therefore provide only a descriptive analysis of the data by reporting numbers rather than risks. We trust that all patients received thromboprophylaxis according to national or international guidelines, as adherence to guidelines is very high in German speaking countries [18]. However, the duration of pharmacologic prophylaxis was not retrievable from the medical records in some patients. We excluded from the study patients with strong thrombophilia whom we regarded as potential candidates for prolonged secondary thromboprophylaxis. Our conclusions therefore do not apply for this patient population.

## Conclusion

In conclusion, among patients with a first unprovoked VTE the number of recurrences provoked by a temporary risk factor appears to be low suggesting that patient counseling and thromboprophylaxis is adequate and effective. VTE often recurs at a time when preventive measures are already stopped or even during thromboprophylaxis. Surgery and trauma are the most frequent risk factors. Recurrent events can most likely be prevented by prolonged or intensified prophylaxis, but presumably at the expense of a higher bleeding risk.

## Abbreviations

VTE: Venous thromboembolism
DVT: Deep-vein thrombosis
PE: Pulmonary embolism
IQR: Interquartile range
